# Sutureless Adult Voluntary Male Circumcision with Topical Anesthetic: A Randomized Field Trial of Unicirc, a Single-Use Surgical Instrument

**DOI:** 10.1371/journal.pone.0157065

**Published:** 2016-06-14

**Authors:** Justin Shenje, Peter S. Millard

**Affiliations:** 1 Department of Epidemiology, School of Public Health and Family Medicine, University of Cape Town, Cape Town, South Africa; 2 Department of Epidemiology, Graduate Program in Public Health, University of New England, Portland, Maine, United States of America; Johns Hopkins University Bloomberg School of Public Health, UNITED STATES

## Abstract

**Introduction:**

The World Health Organization has solicited rapid and minimally invasive techniques to facilitate scale-up of voluntary medical male circumcision (VMMC).

**Study design:**

Non-blinded randomized controlled field trial with 2:1 allocation ratio.

**Participants:**

75 adult male volunteers.

**Setting:**

Outpatient primary care clinic.

**Intervention:**

Open surgical circumcision under local anesthetic with suturing vs. Unicirc disposable instrument under topical anesthetic and wound sealing with cyanoacrylate tissue adhesive.

**Primary Outcome:**

Intraoperative duration.

**Secondary Outcomes:**

Intraoperative and postoperative pain; adverse events; time to healing; patient satisfaction; cosmetic result.

**Results:**

The intraoperative time was less with the Unicirc technique (median 12 vs. 25 min, p < 0.001). Wound healing and cosmetic results were superior in the Unicirc group. Adverse events were similar in both groups.

**Conclusions:**

VMMC with Unicirc under topical anesthetic and wound sealing with cyanoacrylate tissue adhesive is rapid, heals by primary intention with superior cosmetic results, and is potentially safer and more cost-effective than open surgical VMMC.

**Trial Registration:**

Clinicaltrials.gov NCT02443792

## Introduction

Voluntary medical male circumcision (VMMC) reduces female-to-male HIV transmission and is a key element in the UNAIDS plan to end the global AIDS epidemic by 2030.[1] Cultural and organizational challenges remain, and the World Health Organization (WHO) has solicited improvements in VMMC techniques as an essential ingredient to enhance VMMC scale-up.[2]

The challenge is to develop circumcision methods suited to resource-limited settings and which can be more easily adopted by mass circumcision campaigns without compromising patient safety. The WHO recently approved two plastic ring devices, one of which is increasingly used in HIV prevention programs.[3] While these devices require no sutures and have few significant adverse events, the major drawback is they remain on the penis for one week, resulting in necrosis at the base of the foreskin; consequently, healing is prolonged and occurs by secondary intention.

The Unicirc surgical instrument uses an approach fundamentally different from plastic ring devices and functions identically to the Gomco clamp used in early infant circumcision. It is a single-use-only metal and plastic disposable surgical instrument, now in its second version.[4, 5] The Unicirc creates circumferential compression of the base of the foreskin, which fuses the mucosal and skin surfaces and eliminates bleeding after the foreskin is excised. The fused skin edges are sealed with cyanoacrylate adhesive to promote healing by primary intention. The procedure does not require injectable anesthetic or sutures.

The objective of this randomized field trial was to compare the minimally invasive Unicirc technique to conventional open surgical circumcision for important clinical outcomes and adverse events.

## Methods

This study was designed and conducted according to the guidelines found in WHO’s Framework for Clinical Evaluation of Devices for Adult Male Circumcision[2] (Framework).

### Trial design

This was a single-center non-blinded, parallel randomized controlled trial with 2:1 (Unicirc: Surgical) allocation ratio in balanced blocks of 15.

The South African Medical Association’s Ethics Committee (SAMAREC) approved the study and the informed consent. We obtained written informed consent from each participant. All participants were adults. The procedures took place on four dates between July 15 and August 7, 2015. The ClinicalTrials.gov identifier is NCT02443792.

### Participants

Healthy uncircumcised men at least 16 years of age (the age of consent in S. Africa) were eligible for the study. Participants were recruited via posters and word of mouth. Men were recruited from 3 clinics associated with Andrew Saffy Memorial hospital, serving the Lonmin group of platinum mines, situated in Rustenberg, North West, South Africa.

We excluded volunteers with current illness, bleeding disorder, reaction to local anesthetic, infection, or any penile abnormality potentially complicating circumcision.

All subjects had an HIV test performed as per hospital policy, and were referred appropriately. No recruited volunteer was excluded because of HIV status and investigators were unaware of HIV status.

We asked participants to abstain from sexual intercourse for at least 4 weeks after the circumcision and until the wound was completely healed. Condoms were made freely available.

### Intervention

Two generalist doctors, experienced in surgical circumcision, performed all circumcisions assisted by registered nurses in individual consultation rooms in a primary health care clinic.

#### Open surgical technique

After injecting a mixture of lidocaine 2% and bupivacaine as a subcutaneous ring block at the base of the penis, circumcisions were performed with the forceps-guided technique (RL) and the dorsal slit technique (LIM), as described in the WHO’s Manual for Male Circumcision under Local Anesthesia.[6] After suturing with absorbable sutures, the wound was covered with an absorbent gauze dressing.

#### Unicirc with cyanoacrylate skin adhesive

Training on the Unicirc method consisted of demonstrations on a medical model, assisting several cases and then performing at least five cases successfully under supervision before being certified as competent.

The surgical field was cleansed with povidone iodine and 5 gm of topical Lidocaine and prilocaine (EMLA™) was applied to the glans and foreskin 30 minutes prior to procedure. Sizing was determined using a disposable sizing plate similar to that used for plastic ring devices. Prior to use, the instruments were gas sterilized in sealed packages. The Unicirc instrument was applied to the glans; the foreskin was then pulled over the transparent bell with the surgeon’s fingers and adjusted to ensure adequate removal of foreskin. No surgical instruments were used to position the instrument. The Unicirc was then screwed tightly and left in place for 5 minutes before the foreskin was excised with a surgical scalpel. The Unicirc was removed and the fused skin-mucosal edges were sealed with cyanoacrylate skin adhesive (Derma-Flex QS™). Operative time was counted from the time the procedure was begun until the dressing was applied (i.e. including the 5 minute waiting time). There were four different Unicirc diameters used in the study: 2.6 cm, 2.9 cm, 3.2 cm, and 3.5 cm.

We covered the wound with Hypafix™ adhesive dressing and absorbent gauze. We instructed participants to keep the wound dry, to leave the gauze in place until it became soiled, and to remove the Hypafix™ after 3 weeks if it was still in place.

We observed participants for 30 minutes after the procedure, and gave them written postoperative instructions with cellular telephone contact information of the doctor.

### Outcomes

#### Primary Outcome Measure

Intraoperative duration.

#### Secondary Outcome Measures

Intraoperative and postoperative pain; adverse events (intraoperative and postoperative); wound disruption, healing at 4 weeks; patient satisfaction; and, cosmetic result.

#### Adverse events

The Framework served as the guideline for evaluating adverse events (AEs). Mild AEs required little or no intervention (e.g. slight bleeding), moderate AEs required active treatment (e.g. antibiotics or suturing), and severe AEs required transfusion or hospitalization, or resulted in permanent damage. Key AEs evaluated were anesthetic complications, bleeding, hematoma, infection, problems with urination, subsequent procedures conducted to correct complications or poor cosmetic results, and occupational exposure.

### Sample Size

We based the sample size on the Framework which states: “Studies involving about 100 men (range 50 to 300) are suggested as a compromise between assessing safety, documenting the presumed advantages of the new method, and ensuring rapid progress through the different stages of clinical assessment. For devices which are aids to surgery and do not stay on the penis….study sizes at the lower end 25–100 range may be sufficient.”

The sample size of 75 gave >90% power to detect a mean difference of 10 minutes in duration of surgery.

### Randomization and implementation

An investigator who was not involved in the surgeries allocated participants in a 2:1 ratio using a random number table; block randomization (in blocks of 15) was used to ensure exact 2:1 allocation. Each assignment was written on a slip of paper, folded, and placed in a sealed, opaque envelope. Each envelope was opened only at the time of placement of the anesthetic.

### Follow-up

Follow-up was at seven days and four weeks. If the wound was not completely healed by four weeks, we conducted a six-week follow-up visit.

### Outcome assessment

Outcome definitions are shown in Table 1. Intraoperative duration included the 5 minutes waiting time after applying the Unicirc. The surgeons themselves assessed wound healing outcomes.

**Table 1:** Outcome definitions.

Outcome	Definition
**Pain assessment using Visual Analog Scale (0–10)**	Self-reported intraoperative and immediate postoperative pain
**Blood loss**	Estimated by surgeon (ml)
**Intraoperative duration**	From the moment the procedure was started until dressing placed
**Adverse Event**	Mild adverse events: no active intervention other than wound pressure for bleeding. Moderate events: medical intervention (sutures, antibiotics). Severe events: transfusion; hospitalization; or resulted in permanent disfigurement
**Wound infection**	Wound swelling, redness, pain, purulent discharge
**Wound disruption**	Length of wound disruption (< 2cm vs. > 2cm)
**Wound fully healed**	Wound completely closed. No superficial ulcerations or granulation tissue present
**Cosmetic appearance**	Regular: scar line straight with no irregularity
	Irregular: Some irregularity to scar line
	Scalloped: wavy appearance to scar line
**Participant satisfaction (5 point Likert scale)**	Are you satisfied with your circumcision result?

We used a 10-point visual analog scale for the intraoperative and postoperative pain survey, which was administered just prior to leaving the clinic. Satisfaction was assessed at the final visit using a 5-point Likert scale.

### Data analysis

Analysis was by intention-to-treat. We collected data from participants on socio-demographics and reason for circumcision. HIV status was not recorded as part of the study protocol. We analyzed data with Epi Info, version 7 (Atlanta, USA). For continuous variables, we used the nonparametric Mann-Whitney/Wilcoxon two-sample test for comparison of outcomes. For categorical variables, we used the chi-square or Fisher’s exact test.

## Results

### Participant flow

**Fig 1:**
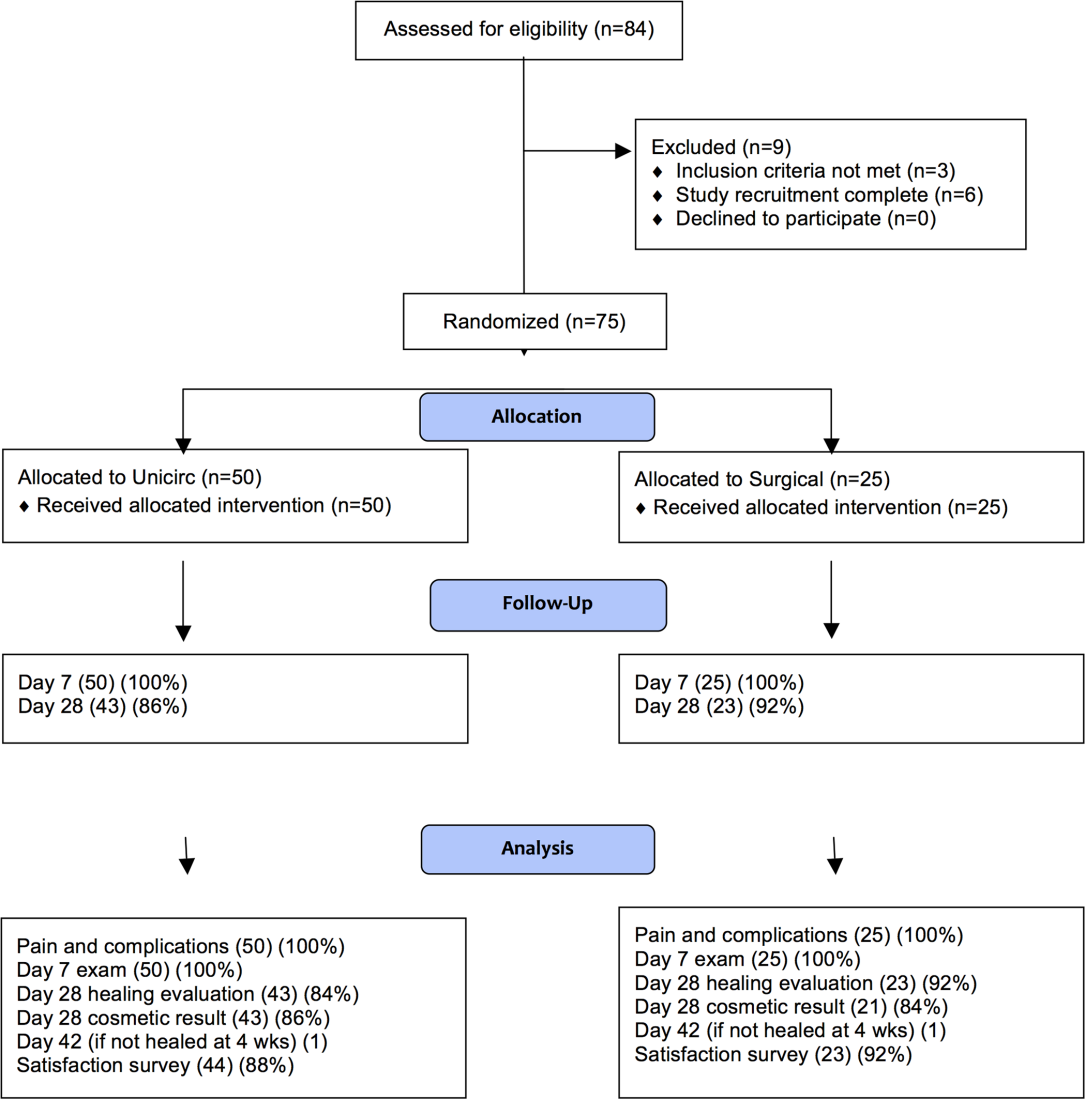
Participant Flow Diagram.

We recruited participants during May and June, 2015. Followup was completed in September, 2015. Although subjects at least 16 years of age were eligible, all participants were employees of the mining company and were at least 18 years of age. The flow of participants in the study is shown in Fig 1. A total of 84 men were interviewed and 75 (89%) participated in the study. All participants received the intervention to which they were randomly assigned. One doctor (LIM) performed 42 circumcisions and the other (RL) performed 33 circumcisions, each approximately proportionately distributed between the Unicirc and surgical techniques.

### Baseline data

The baseline characteristics of the participants are shown in Table 2. Most men gave improved hygiene as their motivation for circumcision; one-third were motivated by a potential reduction in HIV infection. There were no significant differences in baseline characteristics between the two groups.

**Table 2:** Baseline characteristics.

	Unicirc (N = 50)	Open surgical (N = 25)
**Age (yrs), n (%)**		
* 18–25*	3 (6)	2 (8)
* 26–35*	19 (38)	12 (48)
* 36+*	28 (56)	11 (44)
***Median age (yrs)***	37.5	34
**Marital status, n (%)**		
* Married*	29 (58)	9 (36)
* Single in a relationship*	19 (38)	16 (64)
* No partner*	2 (4)	0
**Religion, n (%)**		
* Christian*	29 (58)	20 (80)
* Muslim*	0	0
* African Independent*	2 (4)	0
* No religion*	10 (20)	2 (8)
* Other*	9 (18)	3 (12)
**Highest educational level, n (%)**		
* No primary*	2 (4)	2 (8)
* Primary*	10 (20)	1 (4)
* Secondary*	38 (76)	21 (84)
* Post-secondary*	0	1 (4)
**Reason for circumcision, n (%)**		
* Hygiene*	25 (50)	13 (52)
* Reduce HIV infection*	14 (28)	10 (40)
* Appearance*	3 (6)	0
* Social/religious*	1 (2)	0
* Sexual pleasure*	3 (6)	2 (8)
* Other*	4 (8)	0

### Outcomes analyzed

Table 3 shows operative outcomes. Intraoperative and postoperative pain was minimal in both groups. Intraoperative duration and blood loss were less with the Unicirc method, median duration 12 vs. 25 min (p < 0.001) and median blood loss 1.5 vs 40 ml (p< 0.001).

**Table 3:** Operative outcomes.

	Unicirc	Open surgical
Operative duration (min), median (IQR)*	12 (11,17)	25 (21,35)
Estimated blood loss (ml), median (IQR)*	1.5 (1,2)	40 (40,50)
**Intraoperative Pain (10 point scale), median (IQR)**	1.0 (0.5,2)	1.0 (0,2)
**Postoperative pain (10 point scale), median (IQR)**	1.0 (0.5,2)	1.0 (0,5)

IQR = Interquartile range

*P < 0.001

Table 4 shows adverse events. Two intraoperative complications occurred using the Unicirc instrument. In one case, the doctor neglected to tighten the Unicirc; in the second case, the Unicirc was mis-packaged by staff and the size of the bell did not match the size of the yoke, resulting in inadequate compression. Each of the two procedures was completed surgically; both were analyzed in the Unicirc group to which they were assigned. There was one bleeding episode, which occurred two hours following a Unicirc procedure and was sutured. None of the 3 hematomas in the surgical group required intervention. There were no significant differences between the two groups in bleeding, hematoma, infection, or any other complication.

**Table 4:** Adverse events.

	Unicirc	Open surgical
**Intraoperative suturing, *n* (%)**	2 (4)	All by protocol
**Serious postoperative complication, n**	0	0
**Postoperative bleeding, n (%)**		
* Mild (dressing only)*	0	1 (4)
* Moderate (sutured)*	1 (2)	0
**Hematoma, n (%)**	0	3 (12)
**Postoperative infection *(requiring antibiotic)*, n (%)**	0	0

P > 0.05 for all comparisons

Healing, participant satisfaction, and cosmetic results are shown in Table 5. Eighteen subjects [14 Unicirc (28%) and 4 surgical (16%)] experienced minor (< 2 cm length) wound disruptions at the one-week followup visit. The Unicirc wound disruptions were accompanied by loosening of the Hypafix™, suggesting that they had been chronically wet. No wound disruptions required intervention, all healed uneventfully, and we did not consider them adverse events.

**Table 5:** Postoperative outcomes.

	Unicirc	Open surgical	P value
**Wound disruption at 1 wk, n (%)**			
*< 2 cm length*	14 (28)	4 (16)	NS
*> 2 cm length*	0	0	
**Wound fully healed *at 4 weeks*, n (%)**	39 (90.7)	16 (69.6)	0.04
**Satisfaction, n (%)**			
*Very satisfied*	43 (97.7)	22 (95.7)	NS
*Satisfied*	1 (2.3)	1 (4.4)	
*Not satisfied*	0	0	
**Cosmetic results, n (%)**			
*Regular*	40 (93.0)	2 (9.5)	P < 0.001
*Irregular*	3 (7.0)	6 (28.6)	
*Scalloped*	0	13 (61.9)	

One of the seven subjects in the Unicirc group who did not return for the 28-day followup visit was successfully contacted by phone to administer the satisfaction survey. Unicirc subjects were more likely to be fully healed at 4 weeks (90.7% vs 69.6%; p = 0.04). Satisfaction was high and equal in both groups. The cosmetic result was superior in the Unicirc group; a regular scar line was found in 40 (93.0%) of the Unicirc subjects vs. 2 (9.5%) in the surgical group (p < 0.001).

## Discussion

Scale-up of VMMC to prevent HIV infection in Africa has now exceeded 9 million men, which is well below WHO’s goal of 20.8 million men in 14 high priority African countries by 2016.[7] In order to more effectively scale-up services, we require fundamental improvements in circumcision techniques and programs.

This study showed that, compared to surgical VMMC, the Unicirc method had a shorter procedure time, more rapid healing, better cosmetic appearance, and similar adverse events.

The generalist doctors in this study were highly experienced in open surgical circumcision but had not previously used the Unicirc instrument outside of training. Operative duration was longer than in previous studies,[4, 5] but Unicirc required approximately half the operative time compared to the surgical technique. We believe that the relatively long operative times were the result of the surgical style of the two participating doctors. We expect even greater time-savings as the generalist doctors gain more experience with the Unicirc method. Because the Unicirc operative duration includes 5 minutes of waiting for the tissues to fuse, multiple procedures can be performed simultaneously using the WHO’s MOVE model of task-sharing.[8]

Unicirc requires no surgical instruments other than a scalpel, no injectable anesthetic, and no sutures. The retail cost of a sterile pack for a Unicirc circumcision is US$5 versus US$20 for the surgical pack, Derma-Flex QS US$15.50, and EMLA cream US$4.30 in South Africa. The cost of the Unicirc has not been determined but will likely be similar to plastic ring devices. Men can return to work the next day and do not need to take a day off work for a one-week device removal visit. Because there is never an open wound, Unicirc can be performed as a clean, rather than a sterile, procedure. These factors and the substantial time-savings with Unicirc are likely to substantially reduce overall cost and assist in mass VMMC scale-up. However, a formal economic evaluation is required to quantify the overall cost of the Unicirc method compared to other techniques.

Adverse events were low with no significant differences between groups. There were 2 intraoperative complications with Unicirc, one related to surgical inattention and one related to faulty packaging by staff. The Unicirc kit is now pre-packaged at the factory to prevent mismatching of parts.

One Unicirc participant experienced postoperative bleeding which required suture. In the first study with this instrument, there were no postoperative bleeds in 50 subjects;[5] in the second study, postoperative bleeding occurred in 5 (4.5%) subjects.[4] As in the prior studies, there were no hematomas with Unicirc. Because of the small risk of postoperative bleeding, we recommend that sites capable of suturing perform Unicirc circumcisions.

This study showed superior healing and cosmetic results using the Unicirc method. Absorbable sutures frequently cause small ulcerations at the entry and exit points, which probably accounted for the differences in healing. There were 28% minor wound disruptions following Unicirc (vs. 16% following surgical), but they were not clinically important and did not delay overall healing.

After numerous paediatric[9–14] and adult[4, 5, 15–20] studies, there remains no doubt that cyanoacrylate tissue adhesive is superior to sutures in VMMC. We used high viscosity, quick-setting 2-octyl cyanoacrylate, which applied easily and cured quickly. The one clear caveat is that the wound must be kept dry postoperatively; nothing adheres to macerated skin.

The use of topical anesthetic, coupled with this sutureless technique, will reduce the risk of needlestick injuries and therefore the transmission of blood borne infections.

This field study was unblinded and was performed at a single center by two doctors. Wound healing was assessed by the doctors themselves and there was no independent assessment. Followup at four weeks was not complete, but we feel that all adverse events were accounted for, since followup at 7 days was complete and almost all VMMC complications occur in the first week. The study was underpowered to evaluate adverse events, but neither this nor prior Unicirc studies have shown elevated adverse event rates compared to other methods.

Two plastic ring devices have been approved by WHO and are now being used in HIV prevention programs. However, healing is delayed by secondary intention, so these devices do not meet the criterion laid out in WHO’s Framework that new devices have “more rapid healing than current methods and/or might entail less risk of HIV transmission in the immediate postoperative period.”[2] Furthermore, the fact that the placement of plastic ring devices is faster than surgical circumcision is misleading, because the necessity for a second visit for device removal nullifies any time advantage. There is direct evidence of HIV shedding from unhealed circumcision wounds in HIV-infected men at 3 weeks, suggesting caution in using plastic ring devices because of delayed healing by secondary intention.[21] It is also likely that increased HIV acquisition may occur among men who have sex with HIV-infected partners prior to complete healing of circumcision wounds. Tetanus is probably more likely to occur with plastic ring devices than other methods due to the presence of necrotic material.[22]

## Conclusions

VMMC with Unicirc under topical anesthetic and wound sealing with cyanoacrylate tissue adhesive is rapid, heals by primary intention, has superior cosmetic results, and is potentially safer than open surgical VMMC.

## Supporting Information

S1 CONSORT Checklist Sutureless adult voluntary male circumcision with topical anesthetic: a randomized field trial of Unicirc, a single-use surgical instrument. (DOC)

S1 Protocol Sutureless adult voluntary male circumcision with topical anesthetic: a randomized field trial of Unicirc, a single-use surgical instrument. (PDF)
