# Supplementary material for: Sutureless Adult Voluntary Male Circumcision with Topical Anesthetic: A Randomized Field Trial of Unicirc, a Single-Use Surgical Instrument
Source: PLoS One. 2016 Jun 14;11(6):e0157065. doi: 10.1371/journal.pone.0157065 (PMC4907451; doi:10.1371/journal.pone.0157065)
Supplement: S1 Protocol — (PDF) [file pone.0157065.s002.pdf]

S2 Protocol. Sutureless adult voluntary male circumcision with topical anaesthetic: a randomized field trial of Unicirc, a single-use surgical instrument

Principal Investigator: Justin Shenje, MB ChB

Co-investigator: Peter Millard, MD, PhD

Sponsor: Simunye Primary Health Care

SUMMARY

According to the Framework for Clinical Evaluation of Devices for Adult Male Circumcision (WHO, 2011): *"WHO and other health authorities wish to identify one or more devices that (a) would make the VMMC safer, easier, and quicker; (b) would have more rapid healing than current methods and/or might entail less risk of HIV transmission in the post-operative period; (c) could be performed safely by health-care providers with a minimal level of training; and (d) would be cost-effective compared to standard surgical methods for male circumcision scale up."*

Because of difficulties and small risks entailed in using sterilizable instruments, WHO prefers disposable, single-use-only instruments be used for circumcision. This proposed randomized controlled trial will provide important data which will inform and enable the South African health system to more effectively scale-up circumcision services. This study will prove that VMMC using the Unicirc disposable single-use-only instrument coupled with tissue adhesive meets WHO criteria for the ideal method: it is faster, easier to learn, safer for both surgeons and patients, heals sooner, has excellent cosmetic results, and is more cost effective than any other currently available technique.

We propose a randomized controlled trial (RCT) comparing this minimally-invasive circumcision technique to the open surgical technique. In accordance with WHO recommendations concerning sample size found in the Framework for Clinical Evaluation of Devices for Adult Male Circumcision, we propose to study:

- Unicirc disposable instrument with topical anaesthetic and tissue adhesive wound sealing: 50 men

- Open surgical circumcision: 25 men

This is the fourth study of Unicirc circumsion, and the second study that uses topical anaesthetic instead of injectable local anaesthetic. Tissue adhesive is widely used in multiple areas of medicine; specifically, multiple observational studies and RCTs have shown cyanoacrylate tissue adhesives to be superior to suture closure in VMMC. The open surgical method will serve as the control intervention.

Population: Men at least 18 years of age who desire male circumcision

Study design: Randomized controlled trial

Intervention: Unicirc instrument circumcision plus tissue adhesive vs. open surgical circumcision

Follow-up visits: 7 days and 28 days. Optional 42 day follow-up if not completely healed by 4 weeks.

Primary outcome: Intraoperative time

Secondary outcomes: Complications (operative and post-operative); time to healing; direct costs; post- operative pain; patient satisfaction; cosmetic result.

CIRCUMCISION RANDOMIZED CONTROLLED TRIAL

Subject Number :____________

Date when entered study: ____________

Date of Circumcision: ____________

Date of follow-up done:	Date of next follow-up visit
1. __________________	__________________
2. _________________	__________________
3. __________________	__________________
4. __________________	__________________

UNICIRC clinical trial 004
Contact Form

Center:	Study Staff Name:	Date of Visit:
	|______________________|	|___|___|/|___|___|/| 2 | 0 | 1 |___| Day Month Year

Participant Contacts

Participant Name ____

Study Number __________

Mobile phone number____

Second phone number ____

Address ____

____

____

____

Name of a family member to be contacted in case of emergency

____

Phone number of same family member ____

UNICIRC 4

Brief Eligibility Interview

Center:	Study Staff Name:<br>|__________________|	Today's Date:<br>|___|___|/|___|___|/|_2_|_0_|_1_|___| Day Month Year

Must all be YES to participate?

- Requests circumcision and would potentially like to be part of a study? Yes ☐ No ☐
- At least 18 years of age? Yes ☐ No ☐
- Able to provide informed consent to participate? Yes ☐ No ☐
- Willing to participate in 2 follow-up visits? Yes ☐ No ☐
- Generally healthy? Yes ☐ No ☐

Must all be NO to participate?

- Any current significant illness? Yes ☐ No ☐
- Experiencing urethral discharge? Yes ☐ No ☐
- Currently taking aspirin? Yes ☐ No ☐
- Past reaction to local anesthetic? Yes ☐ No ☐
- Known bleeding disorder or tends to bleed profusely when cut? Yes ☐ No ☐

Volunteer is potentially eligible to participate **Yes ☐ No ☐**

Flowsheet for Potential Participants

Unicirc 4 Trial

Center:	Participant Number:	Study Staff Name:	Today's Date:
	|___|___|___|	|__________|	|___|___|/|___|___|/| 2 | 0 | 1 |___| Day Month Year

1) Eligibility Interview Inclusion/Exclusion ☐ COMPLETE
   a. ☐ Excluded b. ☐ Included ------------→ Start Informed Consent Process
2) Participant agrees to return for control at 7 days and 4 weeks. ☐ COMPLETE
3) Informed Consent ☐ COMPLETE
   a. Not signed ---------→ Excluded b. Signed -----→ Continue
      - Respond to all of participant's questions.
      - Give the participant a copy of the informed consent
4) Assign the participant number and sign the form ☐ COMPLETE.
5) Fill in the participant's contact info ☐ COMPLETE
6) Initial questionnaire· ☐ COMPLETE
   a. Complete the questionnaire with the participant ----→ Conduct Physical Exam
7) Physical Exam
   a. General exam ☐ COMPLETE AND NO EXCLUSIONS
      i. Genital Exam ☐ COMPLETE AND NO EXCLUSIONS
   b. Schedule circumcision. ☐ COMPLETE

Screening Visit: Demographics

Unicirc 004

Center:	Participant Number:	Study Staff Name:	Today's Date:
	|___|___|___|	|________|	|___|___|/|___|___|/| 2 | 0 | 1 |___| Day Month Year

1. **What is the participant's age?**......... |____|____|

2. **What is the participant's relationship with his primary sex partner (wife or steady girlfriend)?**.................................... |____|
   1 = Married and living with wife
   2 = Married and not living with wife
   3 = Not married with regular partner
   4 = No primary sex partner
   5 = Polygamous
   6 = Other, specify: ________________________
   ____

3. **What is the participant's highest level of academic education?** .... |____|
   1 = No formal education
   2 = Some Primary
   3 = Completed Primary
   4 = Some Secondary
   5 = Completed Secondary
   6 = Post Secondary
   7 = Other, specify: ________________________

4. **What is the participant's religion?** |___|___|
   01 = Christian
   02 = Muslim
   03 = Nomiya
   04 = African Independent Church
   05 = Hindu
   06 = No religion
   07 = Other, specify: ______________________

5. **What is the participant's primary reason for circumcision?**...................... |____|
   1 = Partial HIV protection
   2 = Hygiene
   3 = Social / Religious
   4 = Appearance
   5 = Sexual Pleasure
   6 = Medical, explain: ______________________
   ____
   7 = Other, specify: _______________________
   ____

Initials of person completing form: __________________ Date (DD/MM/YYYY):_________________

Screening Visit: History and Physical
Unicirc 004 Trial

Center:	Participant Number: |___|___|___|	Study Staff Name: |_________|	Today's Date: |___|___|/|___|___|/|_2_|_0_|_1_|___| Day Month Year

VITAL SIGNS, HEIGHT & WEIGHT

1. **Blood pressure (mm Hg):** |___|___|___|/|___|___|___|
2. **Weight in kilograms (kg):** |___|___|___|.|___|

HISTORY

3. **Does the participant have any of the following conditions or complaints? (Mark ☒)**

Condition:	No	Yes
**3a.** Urethral discharge	☐	☐
**3b.** Genital sore (ulcer)	☐	☐
**3c.** Pain on erection	☐	☐
**3d.** Swelling of the scrotum	☐	☐
**3e.** Pain on urination	☐	☐
**3f.** Difficulty in retracting foreskin	☐	☐
**3g**. Hypertension	☐	☐
**3h.** Fever/Chills	☐	☐
**3i.** Chest Pain/Pressure	☐	☐
**3j.** Shortness of Breath	☐	☐
**3k.** Cough (acute or chronic)	☐	☐
**3l.** Abdominal Pain	☐	☐
**3m.** Jaundice	☐	☐
**3n.** Open cuts or bruises	☐	☐
**3o.** Rash (specify) ____________	☐	☐
**3p.** Other (specify): ____________	☐	☐

4. **Does the participant have any medication allergies?…|___| (Include all anesthetic and other drug, and latex allergies)**
   0 = No → **GO TO QUESTION 6**
   1 = Yes

5. **What allergies does the participant have? (Please indicate the reaction if known).**

Allergy:	Reaction:
**5a.** ____________	____________
**5b**. ____________	____________
**5c**. ____________	____________
**5d**. ____________	____________

6. **Did the participant take aspirin in the past week?** ……………… |___|
   0 = No
   1 = Yes

7. **Does the participant have a bleeding disorder or bleed easily?**…………… |___|
   0 = No
   1 = Yes
   If 1 = YES, PARTICIPANT IS NOT ELIGIBLE. END FORM HERE, SKIP TO INITIALS, COMPLETE THE SCREENING CHECKLIST and FINAL STATUS CRFs

8. **Has the participant been treated for any sexually transmitted infections in the last 1 month?**…………|___|
   0 = No
   1 = Yes
   10a. If YES, describe:____________________
   ____

9. **Does the participant have any complaints about or abnormalities of his foreskin?**….. |___|
   0 = No
   1 = Yes
   12a. If YES, describe:____________________
   ____

PHYSICAL EXAMINATION

10. **Are there any significant abnormalities on general genital examination?**……………. |___|
    0 = No
    1 = Yes
    13a. If YES, specify:____________________
    ____

11. **Is the examination of the penis normal?**….. |___|
    0 = No
    1 = Yes
    14a. If NO, specify:____________________
    ____

12. **Is the participant suitable for both methods of circumcision used in this study?**…………|___|
    0 = No
    1 = Yes
    If 0 = NO, COMPLETE SCREENING CHECKLIST and FINAL STATUS CRFs.

Initials of person completing form: ______________ Date (DD/MM/YYYY):______________

Unicirc 004

Center:	Participant Number:<br>|___|___|___|	Study Staff Name:<br>|____________|	Date of Visit:<br>|___|___|/|___|___|/|_2_|_0_|_1_|___| Day Month Year

Surgical Report Form

Date ___________________________

First surgeon's name ___________________________

Second surgeon's name if applicable ______________________________

Name of Participant ______________________

The Participant has:

Yes No

☐ ☐ Allergy to local anaesthetic

☐ ☐ Hemophilia or easy bleeding

☐ ☐ Anemia

☐ ☐ Currently taking aspirin?

☐ ☐ Urethral Discharge

☐ ☐ Genital ulcers or warts

☐ ☐ Difficulty with sexual function or erections

☐ ☐ Painful or difficult urination

☐ ☐ Other problem ____

Informed consent

☐ The participant was informed about possible complications such as bleeding, hematoma (blood clot formation under the skin), infection, post operative pain and swelling, reaction to local anaesthetic, and suboptimal cosmetic appearance.

Participant's signature

____

Unicirc 004

Center:	Participant Number: |___|___|___|	Study Staff Name: |___________|	Date of Visit: |___|___|/|___|___|/| 2 | 0 | 1 |___| Day Month Year

Surgical Report Form

Genital exam:

☐ Normal ☐ Abnormal

Specify if abnormal ____

The surgical procedure

Check box if EMLA cream used ☐

Local anaesthesia: Amount:_____ ml ring block, ____ ml in frenulum and _____ ml additional.

Frenulectomy done: *Yes* ☐ *No* ☐

☐ *Circumcision with Unicirc and tissue adhesive. Estimated blood loss _________ ml.*

Size of Unicirc instrument used ___________

☐ *Open surgical circumcision.* *Estimated blood loss _________ ml*

Total time of circumcision (from first surgical incision or placement of Unicirc until start of dressing):

_____ min.

Notes:

Complications:

Surgeon's signature: _______________________________________

Post-Operative Instructions Unicirc

Participant ID _______________________________

If you have complications or questions, call the Doctor.

Doctor's name and phone contact _______________________________________

Phone number of Medical Centre _______________________________________

• Swelling is normal in the first week, but if the wound hurts or swells more or gets redder, call the doctor.

• Take paracetamol, two tablets every 4-6 hours when needed for pain (up to 8 tablets per 24 hours)

The adhesive tape (Hypafix) should remain in place for 7 to 10 days, until it falls off. If it has not fallen off by 2 weeks, wet the area and remove it gently.

How to care for your wound:

• Leave only the tape as a dressing. The tape should stay dry.

• You may wash briefly but do not soak or scrub the area. To dry, rub gently with a soft towel.

• Avoid ointments. Do not apply creams or lotions, creams, Vaseline or any other product to the wound.

• Do not rub, scratch or pick at the wound.

• Redness, swelling and pain are common at first, and should be less every day. Contact us if there is increased redness, swelling or pain, if the wound is hot to the touch, or if the wound edges reopen or separate.

Follow-up visits	Date
7 days	
4 weeks	
6 weeks (optional)	

Follow-Up Visit: Genital Exam and Wound Healing

Center:	Participant Number: |___|___|___|	Study Staff Name: |______________|	Date of Visit: |___|___|/|___|___|/| 2 | 0 | 1 |___| Day Month Year

Follow-up Visit (7 days, maximum 10 days after circumcision)

1. **Is this a scheduled visit?**........................ |___|
   0 = No
   1 = Yes
   1a. If NO, what is the reason for this unscheduled visit? ______________________

   ____

2. **What is the post-op day?** ………………..|___|___|
   e.g. 07 = Day 7

GENITAL EXAM

3. **Aside from the circumcision wound, are the genitals normal?** .... |___|
   0 = No
   1 = Yes
   3a. If NO, describe: ______________________

   ____

WOUND HEALING

4. **Did the participant have any difficulty keeping the wound clean and dry?** .... |___|
   0 = No
   1 = Yes
   5a. If YES, describe: ______________________

   ____

5. **Is there wound disruption or dehiscence?**... |___|
   0 = None 1 = < 2cm
   2 = More than 2 cm

6. **Is there any swelling?**.............................. |___|
   0 = None 2 = Moderate
   1 = Mild 3 = Severe (if severe, report as Adverse Event)

For Questions 7-11, if response is "moderate" or "severe," complete AE form and take a picture with participant's permission.

7. **Is there any evidence of infection?**......... |___|
   0 = None 2 = Moderate
   1 = Mild 3 = Severe

8. **Is there any bleeding?**.............................. |___
   0 = None 2 = Moderate
   1 = Mild 3 = Severe

9. **Is there a hematoma?**............................... |___|
   0 = None 2 = Moderate
   1 = Mild 3 = Severe

10. **Does the participant report difficulty or pain with urination?** .... |___|
    0 = None 2 = Moderate
    1 = Mild 3 = Severe

11. **Are there any other complications?**....... |___|
    0 = None 2 = Moderate
    1 = Mild 3 = Severe
    13a. If response is 1, 2, or 3, describe complication: ______________________

    ____

12. **Was any medication given to or prescribed for the participant at this visit?**...|___|
    0 = No
    1 = Yes → **LIST MEDICATIONS**

    ____

    ____

Comments:

Protocol, UNICIRC clinical trial 004
Follow-Up Visit: Genital Exam and Wound Healing

Center:	Participant Number: |___|___|___|	Study Staff Name: |______________|	Date of Visit: |___|___|/|___|___|/| 2 | 0 | 1 |___| Day Month Year

4 weeks, and optional 6 week follow-up visit

1. **Is this a scheduled visit?**........................ |___|
   0 = No
   1 = Yes
   1a. If NO, what is the reason for this unscheduled visit? ______________________
   ____

2. **What is the post-op day?**
   ………………………………………......|___|___|
   e.g. 28 = Day 28 42 =(optional) Day 42

GENITAL EXAM

3. **Aside from the circumcision wound, are the genitals normal?** .... |___|
   0 = No
   1 = Yes
   3a. If NO, describe: ______________________
   ____

WOUND HEALING

5. **Did the participant have any difficulty keeping the wound clean and dry?** .... |___|
   0 = No
   1 = Yes
   5a. If YES, describe:
   ____
   ____
   —

6. **Has the wound completely healed (no wound, completely epithelialized)?**
   .... |___|
   0 = No
   1 = Yes → **SKIP REST OF QUESTIONS**

7. **Is there wound disruption or dehiscence?**... |___|
   0 = None 1 = < 2cm
   2 = More than 2 cm

8. **Is there any swelling?**.............................. |___|
   0 = None 2 = Moderate
   1 = Mild 3 = Severe (if severe, complete AE form)

For Questions 9-13, if response is "moderate" or "severe," complete AE form and take a photo with participant's permission.

9. **Is there any evidence of infection?**......... |___|
   0 = None 2 = Moderate
   1 = Mild 3 = Severe

10. **Is there any bleeding?**.............................. |___|
    0 = None 2 = Moderate
    1 = Mild 3 = Severe

11. **Is there a hematoma?**.............................. |___|
    0 = None 2 = Moderate
    1 = Mild 3 = Severe

12. **Does the participant report difficulty or pain with urination?** .... |___|
    0 = None 2 = Moderate
    1 = Mild 3 = Severe

13. **Are there any other complications?**....... |___|
    0 = None 2 = Moderate
    1 = Mild 3 = Severe
    13a. If response is 1, 2, or 3, describe complication: __________________________
    ____

14. **Was any medication given to or prescribed for the participant at this visit?**..|___|
    0 = No
    1 = Yes → **LIST MEDICATIONS**
    ____
    ____

15. **Final Cosmetic Result** ....|___|
    1= Regular
    2= Irregular
    3 =Scalloped (wavy)

Protocol, UNICIRC clinical trial 004
Follow-Up Visit: Genital Exam and Wound Healing

Center:	Participant Number:	Study Staff Name:	Date of Visit:
	|___|___|___|	|_______________|	|___|___|/|___|___|/| 2 | 0 | 1 |___| Day Month Year

4 weeks, and optional 6 week follow-up visit

CIRCUMCISION ACCEPTABILITY

15. **How satisfied are you with the appearance of your circumcision?**… ……………………… |____|
1 = Very satisfied →
2 = Somewhat satisfied→
3 = Somewhat dissatisfied
4 = Very dissatisfied

16a. If 3 or 4, please explain:
____
____

16. Would you recommend circumcision to a friend or family member?…………………………|____|
1 = Recommend very much →
2 = Recommend somewhat →
3 = Not recommend
4 = Not recommend at all

17. **What did you like about the circumcision?**

18. **What did you NOT like about the circumcision or what improvements would you suggest?**

19. **If the participant is either (1) newly identified as being completely healed at this visit, or (2) has an adverse event or is dissatisfied, does the participant permit us to take a picture of his penis?**............ |____|
0 = No
1 = Yes **(take a picture of penis)**

Comments:

ADVERSE EVENT (AE) FORM
UNICIRC 4 Study

Center:	Participant Number:	Study Staff Name:	Date of Visit:
	|___|___|___|	|___________|	|___|___|/|___|___|/| 2 | 0 | 1 |___| Day Month Year

(Each Adverse Event needs a separate form)
Serious Adverse Events need to be reported to study coordinator immediately

1. **Describe the Adverse Event:**

1a. Post-op bleeding…………………………………… ☐
1b. Hematoma……………………………………… ☐
1c. Wound infection……………………………… ☐
1d. Other:…………………………………………… ☐
List other:

____

____

2. **Date of onset**: |___|___|/|___|___|/|___|___|___|___|
day month year

3. **Which circumcision method did the participant undergo?** .... |___|
1 = Unicirc
2 = Surgical

4. **Related to the circumcision?**...................... |___|
0 = Unrelated 2 = Probably related
1 = Possibly related 3 = Definitely related

5. **Was the AE serious?** ………………………… |___|
0 = No (See AE Guide if uncertain)
1 = Yes **(Report within 24 hours of learning of the AE.)**

6. **Highest severity of the AE during study?**... |___|
1 = Mild ➔ **GO TO QUESTION 8**
2 = Moderate
3 = Severe **(If related to study procedure, report within 24 hours of learning of the AE.)**

7. **If the AE is moderate or severe and related to the circumcision, does the participant permit us to take a picture of his penis?**... |___|
0 = No
1 = Yes **(take a picture)**

8. **Was the AE treated?**.................................. |___|
0 = No
1 = Yes
8a. If YES, describe treatment: ______________
____

9. **Was additional suturing needed to treat this AE?**.. |___|
0 = No
1 = Yes
9a. If YES, specify number stitches placed:____

10. **Outcome of the AE**……………………………… |___|
1 = Resolved without sequelae
2 = Resolved with sequelae, specify:____________
____
3 = AE still present at discontinuation/exit
4 = Participant died as a result of this AE
5 = Unknown

11. **Duration of AE greater than 24 hours?**...... |___|
0 = No
1 = Yes

12. **Date of resolution:**
|___|___|/|___|___|/|___|___|___|___|
day month yea

13. **Please list the job title/classification (e.g., surgeon, general doctor, nurse) of each person participating in the treatment of this AE.**
16a. Staff Type 1: ________________________
16b. Staff Type 2: ________________________
16c. Staff Type 3: ________________________

14. **Which supplies were used to treat this AE? (Mark ☒ all that apply.)**
17a. Suture……………………………………… ☐
17b. Bandages…………………………………… ☐
17c. Catheter…………………………………… ☐
17d. Drugs (List below)………… ☐
17e. Drugs: __________________________ ☐
17f. Other: __________________________ ☐

Comments:

UNICIRC 004

Center:	Participant Number:<br>|___|___|___|	Study Staff Name:<br>|_______________|	Date of Visit:<br>|___|___|/|___|___|/| 2 | 0 | 1 |___| Day Month Year

UNSCHEDULED VISIT FORM

1. **What is the reason for this unscheduled visit?**

   ____

2. **What is the post-op day?** ……………..|____|____|

3. **Aside from the circumcision wound, are the genitals normal?** .... |____|

   0 = No
   1 = Yes
   3a. If NO, describe: ________________________

   ____

4. **Did the participant have any difficulty keeping the wound clean and dry?** ………... |____|

   0 = No
   1 = Yes
   5a. If YES, describe: ________________________

   ____

5. **Is there wound disruption or dehiscence?**... |____|

   0 = None 1 = < 2cm
   2 = More than 2 cm

6. **Is there any swelling?**.............................. |____|

   0 = None 2 = Moderate
   1 = Mild 3 = Severe (if severe, report as Adverse Event)

For Questions 7-12, if response is "moderate" or "severe," complete AE form and take a picture with participant's permission.

7. **Is there evidence of infection?**......... |____|

   0 = None 2 = Moderate
   1 = Mild 3 = Severe

8. **Is there bleeding?**.............................. |____

   0 = None 2 = Moderate
   1 = Mild 3 = Severe

9.

Is there a hematoma?.............................. |____|

   0 = None 2 = Moderate
   1 = Mild 3 = Severe

10. **Does the participant report difficulty or pain with urination?** .... |____|

   0 = None 2 = Moderate
   1 = Mild 3 = Severe

11. **Are there any other complications?**....... |____|

   0 = None 2 = Moderate
   1 = Mild 3 = Severe
   13a. If response is 1, 2, or 3, describe complication: ____________________________

   ____

12. **Was any medication given to or prescribed for the participant at this visit?**..|____|

   0 = No
   1 = Yes → **LIST MEDICATIONS**

   ____

   ____

Comments:

PROTOCOL VIOLATION/DEVIATION FORM

Unicirc 004

**Center:**	**Participant Number:**<br>|___|___|___|	**Study Staff Name:**<br>|___________|	**Today's Date:**<br>|___|___|/|___|___|/| 2 | 0 | 1 |___| **Day Month Year**

1. **Description of violation/deviation**: ____

2. **Reasons for violation, if any: (If none ➔ LEAVE BLANK AND GO TO QUESTION 3)**:

3. **Was this a serious protocol violation (consult protocol and project director)?**
   NO ☐ YES ☐

4. **Date violation began:** day ☐☐ /month ☐☐ / year ☐☐☐☐
   (*If date is not clear, consult project manager to determine the appropriate action.*)

5. **Write "1" in box if, in consultation with the project director, the date could not be determined**. ☐

Investigator's signature indicates that he/she has been notified of this violation:

___________________________________ ____________________
Site Principal Investigator's Signature Date
(*If the investigator has not signed this form, the monitor must write a query to inform the investigator of the violation.*)

___________________________________ ____________________
Monitor's Signature Date

UNICIRC 004

Adult Male Circumcision

PARTICIPANT INFORMATION AND INFORMED CONSENT

**Name of Research Study:**	**Unicirc Circumcision Clinical Trial**
**Researchers:**	Dr. Peter Millard, Scientific Coordinator and Epidemiologist<br>Dr Norman Goldstuck, Data Analyst<br>Dr.......................... Principal Researcher
**Sponsor**	Simunye HealthCare

Introduction and General Information

We are asking you to join a research study to compare two methods of voluntary medical male circumcision. The results of this study will give doctors more information to compare the two methods.

We are planning to conduct this research study in South Africa. Overall, we plan to include 75 men, ages 18 and older.

Your Part in the Research Study
If you agree to be in the research study, the doctor will circumcise you using one of two well-established methods. You will be assigned to receive one of these two methods. **This means that you cannot decide which method we will use.**

This study aims to compare the surgical technique that is most commonly used in South Africa to a new technique. Most centers in South Africa use the open surgical method of circumcision, where the foreskin is removed with a scalpel or scissors, then the skin edges are sewed together. The other technique uses a new instrument called a Unicirc disposable instrument. We think the new technique results in less bleeding, has an excellent cosmetic result, and we think it is easier. Instead of using sutures after cutting the foreskin with the Unicirc instrument, we plan to use tissue glue to seal the wound.

UNICIRC 004

Adult Male Circumcision

The two methods are described below:

**Open surgical Circumcision**	**Unicirc Circumcision**
The doctor will inject medicine to numb the penis so you won't feel pain during surgery.	The doctor will use a cream to numb the penis so you won't feel pain during surgery.
After numbing the penis, the surgical circumcision usually takes about 20 minutes.	After numbing the penis, the Unicirc circumcision usually takes about 9 minutes.
The doctor uses sutures/stitches to prevent bleeding and hold the skin together. The sutures are absorbable, so they don't need to be removed.	The doctor uses tissue adhesive to prevent bleeding and hold the skin together. The tissue adhesive disappears on its own, so it doesn't need to be removed.
The procedure is complete after the surgical circumcision	The procedure is complete after the Unicirc circumcision.
You can shower or bathe 2 days after the surgical circumcision.	You can shower 2 days after the Unicirc circumcision, but you should use a plastic bag to keep the wound dry.
You will see a difference in skin color where the foreskin has been cut, which will disappear over time.	You will see a difference in skin color where the foreskin has been cut, which will disappear over time.

UNICIRC 004

Adult Male Circumcision

If you decide to take part in this trial, you will be one of 75 patients. The study will last for up to 4 weeks. You will be asked to visit the Study Doctor twice, as per the following schedule:

1. Procedure
2. First Follow-up: 7 days after procedure
3. Second Follow-up: 28 days after procedure,

At each visit after the procedure, you will undergo the following examination: Inspection of wound for healing, swelling, bleeding and infection.

If you join this research study, we will ask you questions, which will take about 20 minutes. We will ask you about:

- Your medical history;
- Your opinion of the circumcision; and,
- Any pain or other problems you have after your circumcision.

If you agree to be part of this study, **you will not be permitted to choose the procedure** you would prefer.

You should abstain from sex for at least 4 weeks after surgery to allow time for complete healing.

Follow-up
You will be required to come for follow-up visits at 1 week and 4 weeks after the circumcision. If the wound is not completely healed, we will have another follow-up visit 6 weeks after the circumcision.

Photographs
We may ask permission to take pictures of your penis during the research study. We may use the pictures to show how the wound heals. These pictures will not show your face. We will always ask you for permission before we take a picture.

Possible Risks
The small risks of a circumcision are similar regardless of the method used. Risks include pain, bleeding, infection, injury to the penis, or poor appearance. You could have an increased risk of HIV infection if you have sex before the penis is completely healed. Depending on your job, you might not be able to work for a few days after circumcision. There is a rare risk of a local reaction to antiseptic solution and to the numbing injections.

If there is bleeding after the circumcision, we will need to place sutures (stitches).

UNICIRC 004

Adult Male Circumcision

Sutures can cause skin irritation and can cause a suboptimal cosmetic result if they are not placed correctly. Skin adhesive can cause problems with skin sticking together where not desired if it is improperly placed. Occasionally, it can also cause skin irritation.

Common side effects from a circumcision are swelling, bruising, and pain when the penis is erect. Sometimes, the wound can separate and require suturing later. The risk of wound separation is about the same using both techniques. Just as in any surgical procedure, the wound can become infected and can require use of antibiotics and wound care.

If needed, we will give you medicine or provide other care to make any side effect less serious or hurt less. Most side effects go away within the first few days after circumcision. Very rarely, side effects can be serious, long lasting, or permanent. However, we cannot predict all possible side effects.

Some questions that we ask might be embarrassing. It's ok to refuse to answer such questions.

Other than the general benefits of having a circumcision, as identified by you, there are no other benefits to you from participating in this study.

Who will perform the procedure?
The procedure will be performed by a qualified medical doctor who is experienced in performing circumcisions.

Will I be given post-operative instructions?
Yes, you will be given complete verbal and written post-operative instructions and the phone number of the doctor to call if you have any concerns.

What choices do you have other than being in this study?
You do not have to join this research study if you do not want to. If you join, you can leave at any time.

Can your study participation be stopped even if you don't agree?
The study doctor can take you out of this study without your permission if:

- Continuing in the study could harm you, or
- the study is stopped.

Who will be able to see your medical information?
We will protect all information about you and your taking part in this research study to the best of our ability. We will not use your name in any reports. Information that leaves the clinic will have a code number but not your name. The following groups of people may see your study data and clinical records during and after the study:

- Doctors and staff who conduct the study;
- And the committees that approved this study.

We may use your information for research, legal reasons, or research reports. However, we will **not** use your name in any publications, reports, or presentations about this study.

If you give us permission to take pictures, the pictures we take may appear in educational presentations, but we will never show your name or face. The confidentiality of the photographs will be preserved. They will be stored in a secure locked location and will be shared only with the investigators on this study. No name will be attached to the photos. If the photos are used for educational purposes, no name will be attached to the photos.

If you miss a scheduled visit, we may contact you at home by phone or in person. We will ask you to schedule another visit or let us know if you want to leave the research study. When we try to contact you, we will not say that you are in this research study.

Who will have access to identifiable information related to my participation in this research study?
In addition to the investigators listed on the first page of this consent form and their research staff, the following individuals will or may have access to identifiable information (which may include your identifiable medical information) related to your participation in this research study: Authorized representatives of South African Medical Association Research Ethics Committee may review your identifiable research information (which may include your identifiable medical information) for the purpose of monitoring the appropriate conduct of this research study. No others will have access to identifiable information.

In unusual cases, the investigators may be required to release identifiable information (which may include your identifiable medical information) related to your participation in this research study in response to an order from a court of law. If the investigators learn that you or someone with whom you are involved is in serious danger or potential harm, they will need to inform the appropriate agencies, as required by South African law.

Costs and reimbursement of expenses
You will not be paid for participation in this study. However, a reasonable amount to cover your travel expenses will be paid out to you. Please discuss further details in this regard with the study doctor before commencement of the trial. There will be no costs to you or your medical scheme.

If you have any questions about the study, call Dr. Parker at 0729403741.

If You Have a Health Problem
Call the doctor on ………………… or come back to the clinic right away, at any time during the research study:

- If you notice bleeding from the surgical wound;
- If the pain or swelling at the surgical wound gets increasingly worse;
- If you have difficulty passing urine;

- If you develop a fever within one week of surgery;
- If you have severe pain in your lower abdomen; and/or
- If pus is coming out of the wound.

Insurance and Compensation
The sponsor has obtained insurance for you and the study doctor in the event of trial related injuries. The sponsor assumes no obligation to pay for the medical treatment of other injuries or illnesses not related to the studies. Further detailed information on the payment of medical treatment and compensation due to injury can be obtained from the study doctor should you wish to review it. Any compensation will be paid in accordance with the Association of the British Pharmaceutical Industry (ABPI) Guidelines on Compensation, which guidelines adequately cover the compensation aspects relating to clinical trials. A copy of these guidelines is available from the study doctor on request.

Ethics approval of this trial

SAMAREC follows the standard adopted by the latest version of the American Food & Drug Administration (FDA) and ICH Harmonized Tripartite Guidelines for Good Clinical Practice; and conforms to the guidelines laid down by the World Medical Association, in particular, the Declaration of Helsinki, the Belmont Report, the National Department of Health and the SA Medical Research Council.
SAMAREC reviews research involving human participants, prior to initiation of such research and will focus on the ethical implications of clinical research. Ensuring protection of the rights and welfare of the human participants is the Committee's primary concern. Based on the above-mentioned standards and guidelines, SAMAREC will advise and make suggestions to study doctors where necessary.

Your rights as a participant
If you have any questions about how you are being treated or your rights as a participant, you may contact the following groups:

South African Medical Association Research Ethics Committee	Dr Cyril Parker
Tel: (012) 481 2046	

INFORMED CONSENT

The above information has been explained to me and all of my current questions have been answered. I understand that I am encouraged to ask questions, voice concerns or complaints about any aspect of this research study during the course of this study, and that such future questions, concerns or complaints will be answered by a qualified individual or by the investigator(s) listed on the first page of this consent document at the telephone number(s) given.

UNICIRC 004

Adult Male Circumcision

I understand that I may always request that my questions, concerns or complaints be addressed by a listed investigator. If you have any complaints as a participant, I understand that I may contact the SAMAREC Ethics Committee.

By signing this form I agree to participate in this research study.

I, the undersigned study doctor/health professional, ________________________________ hereby confirm that:

I have read and explained fully, to the participant, named ______________________________ as well as the witness who signed below, with the consent of the patient, the content of this document, indicating the nature and purpose of the trial in which he has volunteered to participate.

I have explained both the possible risks and benefits of the trial and the alternative treatments available.

- The participant has indicated that he/she understands the contents of the document and also that he will be free to withdraw from the trial at any time without giving any reason or jeopardizing his subsequent treatment.
- I have informed the participant on the existence of relevant compensation arrangements in case of an injury attributable to the clinical trial, to which he agrees.
- The participant has had sufficient opportunity to ask questions.
- The participant has voluntarily agreed to participate in this trial.

Participant:

Printed Name	Signature or mark	Date

Study Doctor/Health Professional:

Printed Name	Signature	Date

I, the witness who signed below, confirm that the study doctor has explained fully the content of this document to the participant.

Witness:

Printed Name	Signature	Date

UNICIRC 004

Adult Male Circumcision

Permission to take photographs

I give permission for the study personnel to take pictures of my penis during the research study. These pictures will not show my face. These pictures may be used to show how the wound heals. We will always ask you for permission before we take a picture. It's ok to say no.

____ ____________________

Signature or Thumb Print of Volunteer **Date**
